# Very Strong Hydrogen Bond in Nitrophthalic Cocrystals

**DOI:** 10.3390/molecules29153565

**Published:** 2024-07-29

**Authors:** Kinga Jóźwiak, Aneta Jezierska, Jarosław J. Panek, Andrzej Kochel, Barbara Łydżba-Kopczyńska, Aleksander Filarowski

**Affiliations:** Faculty of Chemistry, University of Wrocław, 14 F. Joliot-Curie Str., 50-383 Wrocław, Poland; kinga.jozwiak@uwr.edu.pl (K.J.); aneta.jezierska@uwr.edu.pl (A.J.); jaroslaw.panek@uwr.edu.pl (J.J.P.); andrzej.kochel@uwr.edu.pl (A.K.); barbara.lydzba-kopczynska@uwr.edu.pl (B.Ł.-K.)

**Keywords:** very strong hydrogen bond, phthalic acid, steric effect, CP-MD, DFT

## Abstract

This work presents the studies of a very strong hydrogen bond (VSHB) in biologically active phthalic acids. Research on VSHB comes topical due to its participation in many biological processes. The studies cover the modelling of intermolecular interactions and phthalic acids with 2,4,6-collidine and N,N-dimethyl-4-pyridinamine complexes with aim to obtain a VSHB. The four synthesized complexes were studied by experimental X-ray, IR, and Raman methods, as well as theoretical Car–Parrinello Molecular Dynamics (CP-MD) and Density Functional Theory (DFT) simulations. By variation of the steric repulsion and basicity of the complex’ components, a very short intramolecular hydrogen bond was achieved. The potential energy curves calculated by the DFT method were characterized by a low barrier (0.7 and 0.9 kcal/mol) on proton transfer in the OHN intermolecular hydrogen bond for 3-nitrophthalic acid with either 2,4,6-collidine or N,N-dimethyl-4-pyridinamine cocrystals. Moreover, the CP-MD simulations exposed very strong bridging proton dynamics in the intermolecular hydrogen bonds. The accomplished crystallographic and spectroscopic studies indicate that the OHO intramolecular hydrogen bond in 4-nitrophthalic cocrystals is VSHB. The influence of a strong steric effect on the geometry of the studied cocrystals and the stretching vibration bands of the carboxyl and carboxylate groups was elaborated.

## 1. Introduction

**Figure 1:**
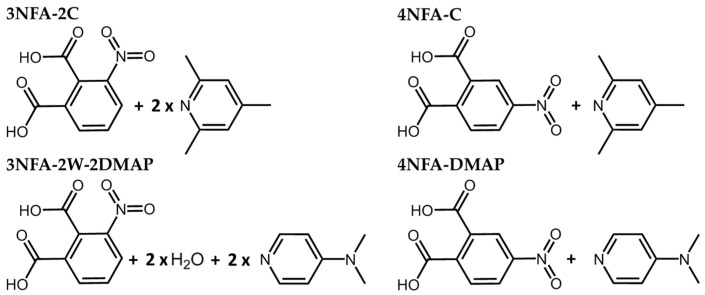
Chemical structures of 3-nitrophthalic acid with 2,4,6-collidine (**3NFA-2C**), 4-nitrophthalic acid with 2,4,6-collidine (**4NFA-C**), 4-nitrophthalic acid with N,N-dimethyl-4-pyridinamine (**4NFA-DMAP**), and 3-nitrophthalic acid–N,N-dimethyl-4-pyridinamine dihydrate (**3NFA-2W-2DMAP**) complexes.

Hydrogen bonds are undoubtedly classified as a vital constituent of biological systems and play an important role in advanced technology [1,2,3,4,5,6,7,8,9,10,11,12,13,14,15]. Its most promising type is a very strong hydrogen bond—a so-called Low-Barrier Hydrogen Bond (LBHB) or Speakman–Hadži hydrogen bond [16,17,18,19]. LBHB takes its term from the extremely low energy barrier on proton transfer or its absence. Notably, the studies of this type of hydrogen bond approved of its essential participation in biological reactions occurring in living organisms [20,21,22,23,24,25]. In the literature, there is a wide discussion as to the prevailing of one or another particular tautomeric form (a proton position) for systems with VSHB [26,27,28]. The neutron diffraction measurements made it possible to map the positional change in the bridging proton, which can be located in the centre of the hydrogen bond [29,30,31,32,33,34,35,36]. As far as LBHB observations are concerned, the compounds with carboxyl groups are of specific interest, especially those with two groups in an adjacent position, like quinolinic acid (2,3-pyridinedicarboxylic acid) [37]. This system features an intramolecular proton transfer of one of hydrogens from the carboxyl group on pyridine and the formation of a very strong intramolecular hydrogen bond [38,39,40]. A number of NMR interesting investigations have dealt with the evaluation of a proton position in the intermolecular hydrogen bond in the complexes of carboxylic acids with pyridine derivatives [41,42,43,44,45,46,47,48]. A promising direction in obtaining a VSHB is the intermolecular transfer of one of the protons of the carboxyl groups of phthalic acid on a pyridine derivative, and, consequently, the formation of a very strong intramolecular hydrogen bond. The importance of the pK_a_ rule for modelling a VSHB should be stressed [49,50,51,52,53]. Supposedly, the studied complexes are characterized by very strong intramolecular and intermolecular hydrogen bonds. This paper concerns obtaining complexes of nitrophthalic acid with pyridine derivatives (Figure 1) and the elaboration of VSHBs, furthering the studies in [54,55]. Experimental (X-ray, IR, and Raman techniques) and theoretical (CP-MD and DFT) methods were used.

## 2. Results

### 2.1. Crystal Structures of the Studied Cocrystals

**Figure 2:**
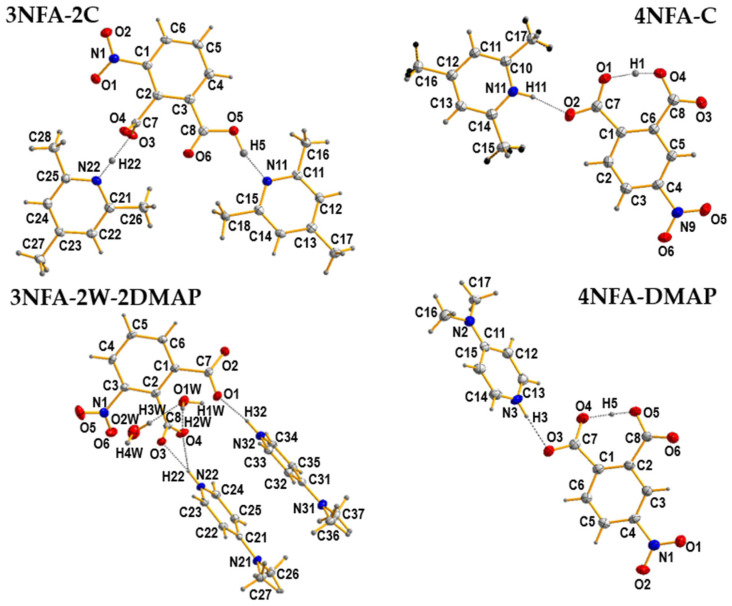
Crystal structures of the **3NFA-2C**, **4NFA-C**, **4NFA-DMAP**, and **3NFA-2W-2DMAP** cocrystals. Hydrogen bonds are denoted with dashed lines. Displacement ellipsoids are plotted at 50% probability level.

The measured crystal structures of the studied cocrystals are shown in Figure 2. The selected X-ray data for hydrogen bonds are listed in Table 1.

### 2.2. Infrared and Raman Spectra of the Studied Cocrystals

**Figure 3:**
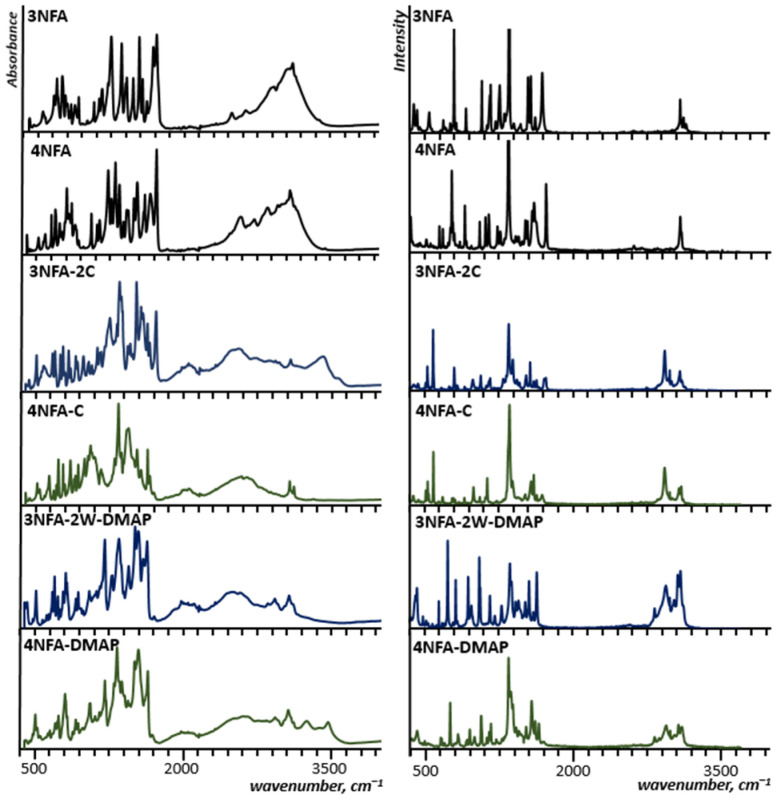
Experimental ATR and Raman spectra of **3NFA**, **4NFA**, **3NFA-2C**, **4NFA-C**, **3NFA-2W-2DMAP**, and **4NFA-DMAP**.

The measured IR and Raman spectra of the studied cocrystals are shown in Figure 3. Notably, IR and Raman spectra of the studied cocrystals do not contain the bands of the **3NFA** and **4NFA** compounds, which testifies to the purity of the obtained cocrystals (1:1 or 1:2 composition). The X-ray results clearly point out the formation of the cocrystals (Figure 2).

### 2.3. CP-MD Simulations of the Studied Complexes

**Figure 4:**
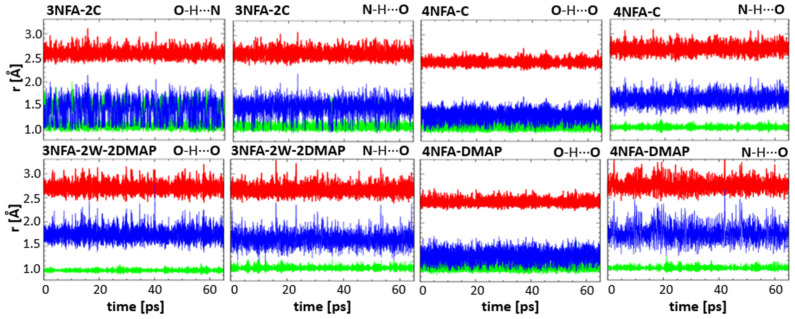
The time evolution of donor–proton (green, d(DH) in Å), proton–acceptor (blue, d(AH) in Å), and donor–acceptor (red, d(DA) in Å) distances simulated by the CP-MD method in the solid state (T = 300 K) for the **3NFA-2C**, **4NFA-C**, **3NFA-2W-2DMAP**, and **4NFA-DMAP** complexes.

The studies of hydrogen bonds of the complexes were accomplished by CP-MD simulations in the solid state. Figure 4 presents the time evolution of the OHO and OHN/NHO hydrogen bond metric parameters (O-H/N-H, H⋯N/H⋯O, and O⋯N/N⋯O distances) for the solid state at 300 K. The X-ray structures were used as starting data for the CP-MD simulations.

## 3. Discussion

### 3.1. Structural Analysis of Hydrogen Bonds in Studied Cocrystals

The components of these complexes—3-nitrophthalic (**3NFA**) and 4-nitrophthalic (**4NFA**) acids as well as 2,4,6-collidine (**C**) and N,N-dimethyl-4-pyridinamine (**DMAP**)—were selected on purpose. The **3NFA** and **4NFA** compounds are characterized by two carboxyl groups in an adjacent position and the nitro group in the ortho and meta positions, respectively (Figure 1). The position of the nitro group enforces the difference between the cocrystals (Figure 2). In the cocrystals with the **3NFA** component, the steric repulsion of the nitro group on the carboxyl or carboxylate group makes it turn at a significant torsional angle. This effect strongly hinders the formation of the OHO intramolecular hydrogen bond. According to the obtained X-ray data in the **3NFA-2C** and **3NFA-2W-DMAP** cocrystals, the carboxylate group, substituted in the ortho position to the nitro group, is located nearly perpendicular to the phenyl ring. As for 2,4,6-collidine and N,N-dimethyl-4-pyridinamine, their basicity governs the protons’ position in the OHN intermolecular hydrogen bonds in the **3NFA** cocrystals (**3NFA-2C** and **3NFA-2W-DMAP**, Figure 2). The **3NFA-2C** cocrystal features the protonation of the only molecule of 2,4,6-collidine, whereas for a stronger base, the protonation of two molecules of N,N-dimethyl-4-pyridinamine is observed.

The **4NFA** cocrystals look different from the **3NFA** cocrystals. The absence of a strong steric repulsion of the nitro group on the carboxyl and carboxylate groups favours the formation of the OHO intramolecular hydrogen bond. A necessary condition for the formation of this bond is the deprotonation of one of the carboxyl groups (the formation of the carboxylate group) by means of either 2,4,6-collidine or N,N-dimethyl-4-pyridinamine. The formed OHO intramolecular hydrogen bond represents a VSHB (Table 1). It is noticeable that the difference in basicity between 2,4,6-collidine and N,N-dimethyl-4-pyridinamine also strongly affects the distance of the OHN intermolecular hydrogen bond in the **4NFA-C** and **4NFA-DMAP** cocrystals. As for a stronger basicity of N,N-dimethyl-4-pyridinamine in the **4NFA-DMAP** cocrystal, a longer OHN intermolecular hydrogen bond is observed compared to the **4NFA-C** cocrystal due to the formation of a NH^+^⋯O^−^ ion pair. As known, the proton transfer and formation of the ion pair elongate a hydrogen bond [7]. Therefore, the intermolecular hydrogen bond in the **4NFA-DMAP** cocrystal is weaker than that in the **4NFA-C** cocrystal because of a stronger basicity of the N,N-dimethyl-4-pyridinamine compared to 2,4,6-collidine. In terms of the influence of the basicity of the pyridine derivatives (2,4,6-collidine pK_BH+_ = 7.43 and N,N-dimethyl-4-pyridinamine pK_BH+_ = 9.7 [56,57]) on the OHO intramolecular hydrogen bond, the increasing basicity leads to a minor reduction in this bond (d(OO) = 2.409 Å in **4NFA-DMAP** < d(OO) = 2.410 Å in **4NFA-C**, Table 1). However, if we compare the studied complexes with the complex of 4-nitrophthalic acid with pyridine (pyridine pK_BH+_ = 5.21), this reduction is clearly noticeable [55]. The hydrogen bond distance (d(OO) = 2.425 Å) in the 4-nitrophthalic acid with pyridine complex [55] is longer than that in the studied complexes (d(OO) = 2.409 Å and 2.410 Å, Table 1). Interestingly, the steric repulsion between the nitro and carboxyl groups plays a major role in the structural design of these complexes [38,39,40,50,58,59,60,61]. If the steric impact in proton sponges [62,63,64], malondialdehydes [65], ortho-hydroxy aryl Schiff bases [66,67], and ketones [68,69,70,71] leads to strengthening of the intramolecular hydrogen bond, then, in the studied 3-nitrophthalic acid complexes, it evokes breaking of the intramolecular hydrogen bond, similarly to some salicylamides [72].

A reliable detector of protons’ position in the hydrogen bond is the distance of the C-O and C=O bonds. According to Glidewell et al. [59], the C-O bond of the C-O-H group (1.317 Å) is 0.1 Å longer than the C=O bond (1.216 Å) in 3-nitrophthalic acid. The X-ray data (Table 2) state that the **3NFA-2C** cocrystal is characterized by the carboxyl (1.325 Å and 1.209 Å) and carboxylate (1.279 Å and 1.222 Å) groups, whereas the **3NFA-2W-DMAP** cocrystal features two carboxylate groups due to the transfer of both protons on **DMAP** (d(CO) = 1.262 Å, 1.242 Å and 1.259 Å, 1.250 Å, Table 2). As for the **4NFA** cocrystals, all CO bonds are no longer than 1.320 Å (even those forming the OHO intramolecular hydrogen bond), and they are carboxylates. It is notable that one of CO bonds of the OHO intramolecular hydrogen formation is longer (d(CO) = 1.301 Å for **4NFA-DMAP** and d(CO) = 1.290 Å for **4NFA-C**) than the other one (d(CO) = 1.268 Å for **4NFA-DMAP** and d(CO) = 1.257 Å for **4NFA-C**). This result shows clearly that the studied OHO intramolecular hydrogen bonds are not centrosymmetric.

### 3.2. Spectral Analysis of Hydrogen Bonds in Studied Cocrystals

**Figure 5:**
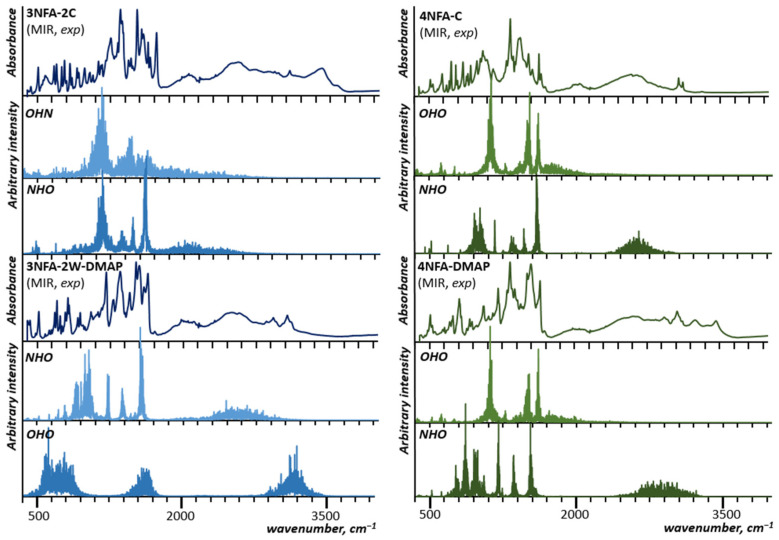
The experimental ATR spectra and atomic velocity power spectra for the bridging protons calculated by the CP-MD method of the **3NFA-2C**, **4NFA-C**, **3NFA-2W-2DMAP**, and **4NFA-DMAP** complexes. The **3NFA** and **4NFA** complexes are presented on the left and right panels, respectively.

To clarify the difference between the formed intra- and intermolecular hydrogen bonds, the measurements and analysis of IR and Raman spectra were completed. For the analysis the most informative bands of the functional groups involved in the hydrogen bond, ν(OH), ν(C=O), and ν_as_(CO_2_^−^) modes were selected. These bands are a good diagnostic tool for the determination of the hydrogen bond strength [3,5,7,73,74,75,76,77,78] and deprotonation of the carboxyl group [79,80,81,82,83]. In a number of papers, the spectral manifestations of the hydrogen bond formation were studied for different carboxylic acids [84,85,86,87,88,89,90,91,92]. The narrow ν(OH) band of the “free” hydroxyl group changes to a broad, intensive, sub-structured band shifted to lower wavenumbers. For spectroscopic studies of the cocrystals, we accomplished the analysis based on IR and Raman measurements (Figure 3), as well as spectra interpretation by the CP-MD method (Figure 5). 

The measured infrared spectra appeared complicated due to the broad bands conditioned by VSHB. These broad bands, abbreviated as *A*, *B*, *C*, and *D* [75] and dependent on the hydrogen bond strength, indicate Zundel continuum absorption [93]. Moreover, the complexity of the observed spectra results from overlapping of at least two broad ν(OH) and ν(NH) bands. Therefore, the assignment of the bands to the corresponding hydrogen bonds was completed by CP-MD simulations for the solid state. This approach allowed us to gain insight into the behaviour of particular atoms via decomposition of the power spectrum of atomic velocity into the atomic components. In the case of protons, such decomposition is particularly valuable because the stretching regions are clearly visible. The methodology of the interpretation of the bands assigned to the hydrogen bond vibrations was applied in papers [54,55].

The CP-MD simulations showed a considerable difference in the positions of the bands assigned to the proton vibrations in the inter- and intramolecular hydrogen bonds. The broad band (2000–800 cm^−1^), assigned to the ν(OH) vibration of the OHO intramolecular hydrogen bond, is visibly red-shifted with respect to the ν(OH/NH) band (2900–2300 cm^−1^), assigned to the OHN intermolecular hydrogen bonds (cf. the OHO spectra with the OHN spectra of **4NFA** complexes in Figure 5). This shift, according to the Badger and Bauer rule [94], confirms the intramolecular hydrogen bond to be much stronger compared to the intermolecular one. This result is in agreement with the accomplished X-ray measurements (Table 1). 

#### 3.2.1. Positions of the ν(C=O) and ν_as_(CO_2_^−^) Bands vs. the Stoichiometry and Geometry of the Studied Cocrystals

The papers [46,79,80,81,82,83] proved the ν(C=O) and ν_as_(CO_2_^−^) bands of carboxyl and carboxylate groups to be the most informative and spectrally sensitive to the formation and stoichiometry of the complexes. Thus, this work deals with the interpretation and analysis of these bands’ positions depending on the geometry and tautomeric form of the complexes. These assignments were completed based on refs. [46,79,80,81,82,83,95,96,97]. 

##### **3NFA-2C** and **3NFA-2W-2DMAP** Cocrystals vs. Their Spectra

**Figure 6:**
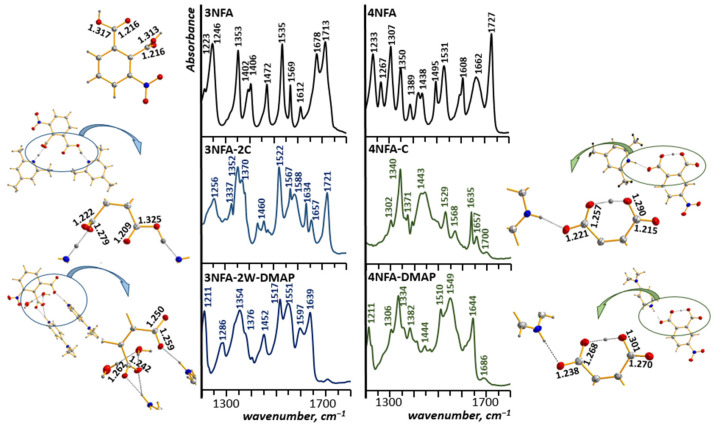
Fragments of experimental ATR spectra and X-ray structures of 3-nitrophthalic acid (**3-NFA**) [56] and 4-nitrophthalic acid (**4-NFA**) as well as 3-nitrophthalic acid–2,4,6-collidine (**3NFA-2C**), 4-nitrophthalic acid–2,4,6-collidine (**4NFA-C**), 3-nitrophthalic acid–N,N-dimethyl-4-pyridinamine dihydrate (**3NFA-2W-2DMAP**), and 4-nitrophthalic acid–N,N-dimethyl-4-pyridinamine (**4NFA-DMAP**) complexes.

Preliminarily, the bands of the **3NFA** compound were analyzed within the range of 1800–1500 cm^−1^ (Figure 6). The spectrum of this compound features bands at 1713 cm^−1^ and 1678 cm^−1^ (spectrum **3NFA** in Figure 6), assigned to the ν(C=O) vibrations of carboxyl groups located in the plane and perpendicular to the phenyl ring, respectively (see crystal structure of **3NFA** ref. [59]). The spectrum of the **3NFA-2C** cocrystal is characterized by two bands in the same range. The band at 1721 cm^−1^ (Figure 6) is assigned to the ν(C=O) vibration of the C(8)O(6)O(5)H(5) carboxyl group, and it is typical for the OH⋯N hydrogen bond without proton transfer (cf. spectra of the **3NFA** compound and spectra of the **3NFA-2C** and **3NFA-2W-DMAP** cocrystals, Figure 6). 

However, two red-shifted bands at 1634 and 1657 cm^−1^ are assigned to the ν_as_(CO_2_^−^) vibrations of the C(7)O(4)O(3) carboxylate group, and, consequently, refer to the hydrogen bonds with proton transfer. The split bands are the result of Fermi resonance between the ν_as_(CO_2_^−^) mode and the low mode overtone [95]. This phenomenon for the acetic acid derivatives with the amines complexes was elaborated by Denisov et al. [96,97]. As for the **3NFA-2W-2DMAP** cocrystal, its spectra in the 1800–1660 cm^−1^ range lack intensive bands, which indicates the absence of carboxyl groups in this complex. The X-ray measurements confirm this result, revealing that both protons of the carboxyl groups were transferred to two **DMAP** molecules, which proves the formation of carboxylate groups. Thereof, the spectrum of the **3NFA-2W-2DMAP** cocrystal contains the ν_as_(CO_2_^−^) bands instead of the ν(C=O) bands within the 1660–1560 cm^−1^ range. Indeed, this range has two intensive bands at 1639 cm^−1^ and 1597 cm^−1^, assigned to the ν_as_(CO_2_^−^) vibrations of the carboxylate groups (see **3NFA-2W-2DMAP** spectrum, Figure 6). The spectrum of the **3NFA-2C** cocrystal is also characterized by an intensive double ν_as_(CO_2_^−^) band, which verifies the presence of the carboxylate group in this cocrystal, as well as the O^−^⋯HN^+^ and O-H⋯N forms. Two bands at 1643 cm^−1^ and 1603 cm^−1^, assigned to the ν_as_(CO_2_^−^) vibration of carboxylate groups of the **3NFA-2W-2DMAP** cocrystal, are conditioned by the different positions of the carboxyl/carboxylate groups with respect to the phenyl ring. Indeed, the X-ray data showed that the C(7)O(2)O(1) carboxylate group is in the plane of the phenyl ring, meanwhile the C(8)O(3)O(4) carboxylate group is placed perpendicularly to this ring. The reason for such geometry of the **3NFA** fragment is a strong steric repulsion between the nitro and carboxylate groups.

##### **4NFA-C** and **4NFA-DMAP** Cocrystals vs. Their Spectra

There are worthy spectral changes in the spectra of the **4NFA** cocrystals without strong steric repulsion between the nitro group and the carboxylate groups. A comparison of IR and Raman spectra of the **4NFA** compound with the spectra of the **4NFA-C** and **4NFA-DMAP** cocrystals exposes the absence of the ν(C=O) bands of the carboxyl groups of the **4NFA** compound (1752 cm^−1^, Figure 6) in the spectra of the **4NFA** cocrystals. However, within the 1700–1600 cm^−1^ range, the spectra of the **4NFA-C** and **4NFA-DMAP** cocrystals possess bands at 1635/1657 cm^−1^ and 1644 cm^−1^, assigned to the ν_as_(CO_2_^−^) vibrations of the carboxylate groups. The results point out the absence of the carboxyl groups and the presence of the carboxylate groups in the **4NFA-C** and **4NFA-DMAP** cocrystals. These spectral observations are supported by X-ray measurements, which show the transfer of one proton from the carboxyl group (the formation of the carboxylate group) on either 2,4,6-collidine (**4NFA-C**) or N,N-dimethyl-4-pyridinamine (**4NFA-DMAP**) and the location of another proton between two carboxylate groups (O^−^⋯H⋯^−^O). These spectral studies are also verified by X-ray measurements, indicating the C-O bond (1.290 and 1.301 Å) in the **4NFA-C** and **4NFA-DMAP** cocrystals to be longer than the C=O bond of the carboxyl groups. The important fact is that all spectral changes described within the 1800–1560 cm^−1^ range are observed in both the IR and Raman spectra. The summary of the archived spectral analysis and the comparison of the spectral characteristics with X-ray data prove that the position of the bands of stretching vibrations of the hydrogen bonds reflects the strength of interactions between the protonodonor and protonoacceptor, whereas the position of the bands of the carboxyl and carboxylate groups forecasts the stoichiometry and location of the proton in the hydrogen bonds.

### 3.3. Potential Energy Curve Calculation for Proton Transfer in Hydrogen Bonds

**Figure 7:**
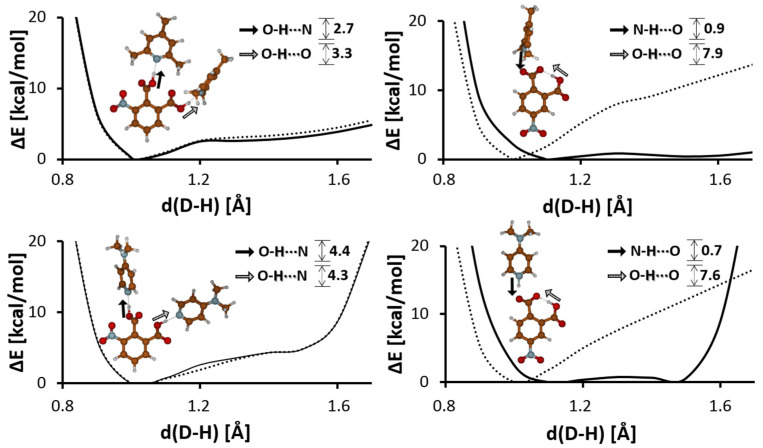
Calculated (B3LYP-D3/6-311+G(d,p)) potential energy curves for gradual elongation of one proton within the inter/intramolecular hydrogen bonds in the **3NFA-2C**, **4NFA-C**, **3NFA-2DMAP**, and **4NFA-DMAP** complexes. The black and white arrows indicate the N-H⋯O and O-H⋯O hydrogen bonds, respectively.

To state if the studied hydrogen bonds are classified as LBHBs, the potential energy curves on proton transfer in the hydrogen bonds were calculated by the DFT method in vacuo. The calculations of the potential curves were made up for the optimized structures of the studied complexes under a gradual elongation of the distance of the O-H/N-H bond at optimization of the rest of structural parameters. When it comes to the **3NFA** complexes, the most stable form is the one with both bridging protons belonging to the carboxyl groups. The calculated potential curves for the **3NFA-2C** and **3NFA-2DMAP** complexes are rather gently sloped under a small barrier (2.7 and 4.4 kcal/mol, Figure 7). The potential curves on proton transfer are pretty similar in both bonds of the complex (see solid and dotted lines, Figure 7). According to papers [98,99,100,101,102,103,104], this picture indicates the possibility of proton transfer in the polar environment (e.g., in the solid state). This fact is approved by the X-ray measurements showing the protonation of 2,4,6-collidine in the **3NFA-2C** cocrystal. A similar trend for the potential curve is typical for the intermolecular hydrogen bond in complexes of nitrobenzoic acid with pyridine or dimethylpyridine [105,106,107]. The potential curves on proton transfer in the OHN intermolecular hydrogen bond in the **4NFA-2C** and **3NFA-2DMAP** complexes is almost symmetrical with two energy minima with a very low barrier (ΔE = 0.7 and 0.9 kcal/mol), which means that this hydrogen bond falls into the LBHB category. As to the intramolecular hydrogen bond in the **4NFA** complexes, the potential energy curve takes a form different from that for the **3NFA** complexes. The most stable structure of the **4NFA** complexes is the form with one proton transferred from the carboxyl group to the nitrogen atom of pyridine derivatives (Figure 7). The potential energy curve on proton transfer in the OHO intramolecular hydrogen bond does not reveal a distinctive second local minimum, though the hydrogen bond is very short. This phenomenon is observed under the matrix isolation condition [108,109] or in solvents at low temperatures [46] with very strong hydrogen bonds, where proton transfer occurs in a number of intermediate states described by potentials with an almost symmetrical single minimum (so-called “mesomeric” scheme [46]).

### 3.4. CP-MD Simulations in Solid State Analysis of Hydrogen Bonds

**Figure 8:**
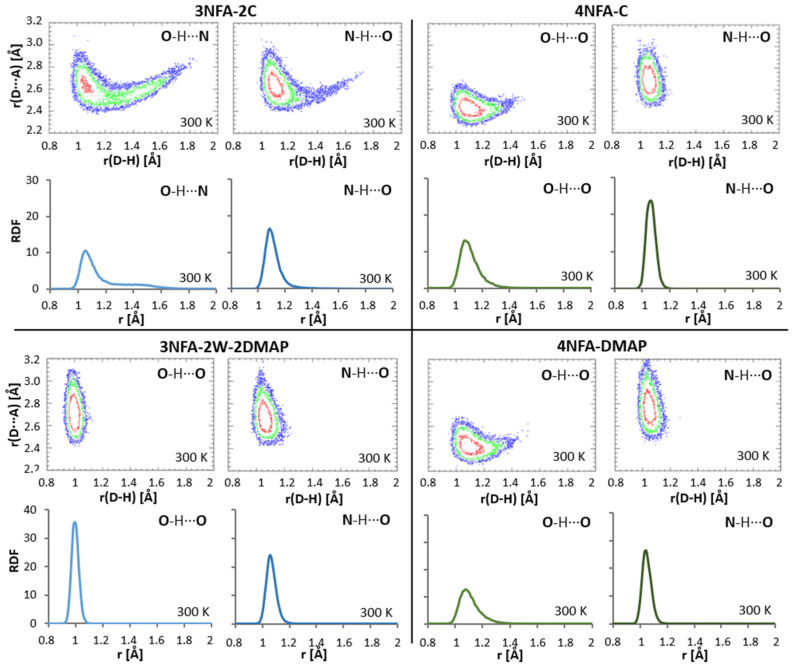
Two-dimensional histograms for the hydrogen atom position in the respective hydrogen bonds obtained by CP-MD simulations at 300 K. Y axes denote the donor–acceptor distances, X axes are the donor–proton distances. Isocontours are drawn at 1 (blue), 5 (green), and 20 (red) Å^−2^ probability density values (**upper** panels). Radial distribution function (RDF) of the studied hydrogen bonds (**lower** panels).

The CP-MD is a very valuable calculation method for the description of the dynamics of hydrogen bonds and interactions between molecules [110,111,112,113,114,115,116,117]. Therefore, an analysis of the dynamics of the hydrogen bond was carried out taking advantage of time evolutions of the interatomic distances (Figure 4) and separate histograms (Figure 8). The “hydrogen bond dynamics” parameter is divided into two components: dynamics of the bridging proton conditioned by the amplitude of the bridging proton vibrations and dynamics of the protonoacceptor–protonodonor bridge (A⋯B). This approach exhibits the interrelation between the bridging protons and hydrogen bridge dynamics. The dynamics of the bridging protons in the studied complexes varies. The dynamics of the bridging proton in the OHO intramolecular hydrogen bond is much stronger than the dynamics of the bridging proton in the O^−^⋯H-N^+^ intermolecular hydrogen bond.

To investigate the dynamics of the hydrogen bonds in the studied complexes, we analyzed the dynamics of the bridging proton (defined by the amplitude of the bridging proton displacements (Δ(H) = d(AH)_max_ − d(AH)_min_)), and the dynamics of the hydrogen bridge (defined by the amplitude of the hydrogen bridge vibrations (Δ(AB) = d(AB)_max_ − d(AB)_min_; where A and B are protonodonors and protonoacceptors, respectively). 

The calculated time-evolution distances (Figure 4) for hydrogen bonds (d(AB), d(AH), and d(BH)) and two-dimensional histograms for the bridging protons (Figure 8) showed significant dynamics (shuttling the bridging proton between the carboxyl group and the nitrogen atom of collidine, Δ(H) = 0.7 Å) of the bridging proton in the **3NFA-2C** complex, whereas in the rest of the complexes Δ(H), they were rather moderate (Δ(H) = 0.2–0.4 Å), i.e., without shuttling the bridging protons. The comparison of the time-evolution distances for the studied hydrogen bonds points out that the dynamics for the intermolecular hydrogen bonds (Δ(AB) = 0.6 Å) are much larger than that for the intramolecular ones (Δ(AB) = 0.4 Å). This difference results from a more rigid O-C-C=C-C-OH fragment closed by a strong intramolecular bond (**4NFA-C** and **4NFA-DMAP**). Interestingly, for the OHO intramolecular hydrogen bond no visible shuttling of the bridging proton between the carboxylate groups was observed, despite the short donor–acceptor distance enforced by the mutual action of the O-C-C=C-C-O covalent skeleton and the strong OHO intramolecular hydrogen bond. The bridging proton in the OHO intramolecular hydrogen bond between the carboxylate groups (COO^−^⋯H⋯^−^OOC) is localized closer to the carboxylate group in the meta position, though the distance between the bridging proton and oxygen of the protonodonor group is relatively large. According to the calculated RDF dependencies (Figure 8), this distance equals about 1.1 Å in both **4NFA-C** and **4NFA-DMAP** complexes. This distance appears to be longer than the hydroxyl group distance for ordinary hydrogen bonds, showing that the OHO intramolecular hydrogen bond is determined by the asymmetric single-well potential. This reflects in extreme red-shifting of the ν(OH) band position analyzed in Section 3.2. Summing up the DFT and CP-MD results concludes that the intramolecular hydrogen bond is strong with a single-well potential energy curve. 

## 4. Materials and Methods

### 4.1. Compounds and Solvent

The 3-nitrophthalic acid, 4-nitrophthalic acid, 2,4,6-collidine, N,N-dimethyl-4-pyridinamine, and methanol were purchased from Merck and used without further purification. The **3NFA-2W-2DMAP** and **4NFA-DMAP** cocrystals were obtained following this procedure: 3-nitrophthalic acid or 4-nitrophthalic acid and N,N-dimethyl-4-pyridinamine (1:1 molar ratio) were dissolved in methanol, and the solvent was slowly evaporated at room temperature. The **3NFA-2C** and **4NFA-C** cocrystals were obtained by dissolution of 3-nitrophthalic acid or 4-nitrophthalic acid in 2,4,6-collidine, and the solution was slowly evaporated at room temperature.

### 4.2. Single Crystal X-ray Structure Determination of Complexes

Crystallographic measurements for the **4NFA-DMAP** and **3NFA-2W-2DMAP** cocrystals were collected with a Κ-geometry diffractometer, Xcalibur Ruby Gemini Ultra, with graphite monochromatized Mo-Kα radiation (λ = 0.71073 Å) at 100(2) K, and the **3NFA-2C** and **4NFA-C** cocrystals were collected with XtaLAB Synergy R, DW system, HyPix-Arc 150 with Cu-Kα radiation (λ = 1.5418 Å) at 100(2) K, using an Oxford Cryosystems cooler. Data collection, cell refinement, data reduction, and analysis were carried out with CrysAlisPro [118] (Table A1). The absorption correction was applied to data with the use of CrysAlisPro. The crystal structures were solved using SHELXT2014 [119] and refined on F^2^ by a full-matrix least squares technique with SHELXL-2016 [120]. Hydrogen atoms were included from the geometry of molecules and difference maps for N–H and O–H. These Figures were prepared using the DIAMOND programme [121]. The data of the cocrystals (CCDC 2302801 (**4NFA-DMAP**), 2301402 (**3NFA-2W-2DMAP**), 2299111 (**3NFA-2C**), and 2299110 (**4NFA-C**)) can be obtained free of charge via www.ccdc.cam.ac.uk/data_request/cif (accessed on 25 July 2024).

### 4.3. Raman and Infrared Measurements

The ATR and Raman (powder) measurements were carried out using Bruker Vertex 70v and Nicolet iS50 spectrophotometers at room temperature with 4 cm^−1^ resolution.

### 4.4. CP-MD in the Crystalline Phase and DFT Calculations

The DFT calculations were carried out with the Gaussian 16 Rev. C01 programme [122]. The Becke functional with Lee–Yang–Parr corrections (B3LYP) [123,124] with a 6-311+G(d,p) basis set [125] was applied for the calculations. The DFT-D3 method [126] was used to reproduce the dispersion forces. The proton reaction path was calculated by lengthening of the OH/NH distance (0.1 Å) with full optimization of the rest of the parameters. The data were visualized with the Molden programme [127]. 

Car–Parrinello Molecular Dynamics simulations were performed using the CPMD programme, version 4.3 [128]. The simulations were carried out in the crystalline phase. The unit cell dimensions are presented in Table A1 and were used as initial parameters for the CP-MD runs. The CP-MD simulations were carried out with periodic boundary conditions and with real-space electrostatic summations for the eight nearest neighbours in each direction (TESR = 8). The PBE exchange-correlation functional [129] coupled with the plane-wave basis set and Troullier–Martins pseudopotentials [130] were used during the molecular dynamics runs. The kinetic energy cutoff for the plane-wave basis set was 100 Ry, while the fictitious electron mass was set to 400 a.u. and the time-step was set to 3 a.u. The temperature applied during the computations was 300 K, controlled by a Nosé–Hoover thermostat chain [131,132]. The time evolution part of the study was divided into two steps: equilibration of the studied cocrystals (50,000 steps; massive thermostatting with a separate Nosé–Hoover thermostat chain for each degree of freedom to ensure fast thermalization; this part of the simulations was excluded from the data analysis), and the production run with standard thermostatting, where the trajectory was collected for ca. 65 ps. The visualization of the obtained results was carried out with the VMD 1.9.3 [133], Mercury [134], and Gnuplot [135] programmes. The spectroscopic properties were extracted from the trajectories using a home-made script: Fourier transform autocorrelation function of atomic velocity power spectra.

## 5. Conclusions

Cocrystals with strong intermolecular and intramolecular hydrogen bonds were obtained. The accomplished X-ray measurements show that the intramolecular hydrogen bonds are very short (d(OO) = 2.410 Å and 2.409 Å). Significant sensitivity of the OHN intermolecular hydrogen bond to the basicity of the pyridine derivatives was shown, whereas the OHO intramolecular hydrogen bond exhibited a weak response. The X-ray results proved that the OHO intramolecular bond in the studied cocrystals is classified as a VSHB, although this bond is asymmetrical. These studies revealed that strong steric repulsion of the nitro group on the carboxylate groups prevents the formation of an intramolecular hydrogen bond in the studied complexes. The CP-MD simulations of the studied cocrystals exposed that the dynamics of the intramolecular hydrogen bond is definitively weaker than the dynamics of the intermolecular one, due to the almost symmetric single-well potential curve on the proton transfer.

## Figures and Tables

**Table 1:** Structural parameters for donor–proton (d(DH)), acceptor–proton (d(AH)), and donor–acceptor (d(DA)) distances (in Å) and the hydrogen bond angle (in °) for the **3NFA-2C**, **4NFA-C**, **4NFA-DMAP**, and **3NFA-2W-2DMAP** cocrystals obtained by X-ray measurements.

Cocrystal	D-H⋯A	Type of HB	d(D-H)	d(AH)	d(DA)	Θ(DHA)
**3NFA-2C**	O(5)-H(5)⋯N(11)	inter	1.07	1.60	2.653(3)	164
	N(22)-H(22)⋯O(3)	-	0.88	1.67	2.543(4)	174
**3NFA-2W-2DMAP**	O(1W)-H(1W)⋯O(2)	inter	0.85	1.97	2.801(2)	165
	O(1W)-H(2W)⋯O(4)	-	0.85	1.90	2.730(2)	167
	O(2W)-H(3W)⋯O(1W)	-	0.85	1.96	2.809(2)	177
	N(22)-H(22)⋯O(3)	-	0.88	1.78	2.655(2)	173
	N(22)-H(22)⋯O(4)	-	0.88	2.54	3.151(2)	127
	N(32)-H(32)⋯O(1)	-	0.88	1.83	2.678(2)	161
**4NFA-C**	O(1)-H(1)⋯O(4)	intra	1.35	1.07	2.410(2)	171
	N(11)-H(11)⋯O(2)	inter	0.88	1.78	2.654(3)	169
**4NFA-DMAP**	O(4)-H(5)⋯O(5)	intra	1.32	1.10	2.409(1)	169
	N(3)-H(3)⋯O(3)	inter	0.86	1.93	2.761(2)	163

**Table 2:** Carboxyl and carboxylate bond distances (in Å) for the **3NFA-2C**, **4NFA-C**, **4NFA-DMAP**, and **3NFA-2W-2DMAP** cocrystals obtained by X-ray measurements.

Cocrystal	Numbering C-O/C=O	Bond Distance d(C-O/C=O)
**3NFA-2C**	C(8)-O(5)	1.325
	C(8)=O(6)	1.209
	C(7)-O(3)	1.279
	C(7)=O(4)	1.222
**3NFA-2W-2DMAP**	C(8)-O(3)	1.262
	C(8)=O(4)	1.242
	C(7)-O(1)	1.259
	C(7)=O(2)	1.250
**4NFA-C**	C(8)-O(4)	1.290
	C(8)=O(3)	1.215
	C(7)-O(1)	1.257
	C(7)=O(2)	1.221
**4NFA-DMAP**	C(8)-O(5)	1.301
	C(8)=O(6)	1.270
	C(7)-O(4)	1.268
	C(7)=O(3)	1.238

## Data Availability

Data are contained within the article.

## Appendix A

**Table A1:** Crystal data and structure refinement for the **3NFA-2C**, **4NFA-C**, **3NFA-2W-2DMAP**, and **4NFA-DMAP** cocrystals.

Crystal Data	CCDC 22999111 (3NFA-2C)	CCDC 2301402 (3NFA-2W-2DMAP)	CCDC 2299110 (4NFA-C)	CCDC 2302801 (4NFA-DMAP)
Empirical formula	C_24_H_27_N_3_O_6_; C_8_H_4_NO_6_, C_8_H_11_N, C_8_H_12_N	C_22_H_29_N_5_O_8_; C_8_H_3_NO_6_, 2(C_7_H_11_N_2_), 2(H_2_O)	C_16_H_16_N_2_O_6_; C_8_H_4_NO_6_, C_8_H_12_N	C_15_H_15_N_3_O_6_; C_8_H_4_NO_6_, C_7_H_11_N_2_
Formula weight	453.48	491.50	332.31	333.30
Temperature	100(2) K	100(2) K	100(2) K	100(2) K
Wavelength	1.54184 Å	0.71073 Å	1.54184 Å	0.71073 Å
Crystal system	Monoclinic	Triclinic	Orthorhombic	Triclinic
Space group	P *2_1_*/c (No.14)	P-1 (No.2)	Pnma (62)	P-1 (No.2)
Unit cell dimensions	a = 7.821(3) Å b = 41.778(3) Å c = 7.253(2) Å β = 109.47(3)°	a = 8.202(3) Å b = 11.125(3) Å c = 13.637(2) Å α = 70.93(4)° β = 85.62(3)° γ = 82.18(3)°	a = 15.8962(5) Å b = 6.6134(3) Å c = 14.6385(5) Å	a = 8.3181(3) Å b = 9.3553(3) Å c = 9.5025(4) Å α = 97.950(3)° β = 92.029(4)° γ = 93.273(3)°
Volume	2234.4(11) Å^3^	1164.4(6) Å^3^	1538.92(10) Å^3^	730.47(5) Å^3^
Z	4	2	4	2
Density (calculated)	1.348 Mg/m^3^	1.402 Mg/m^3^	1.434 Mg/m^3^	1.515 Mg/m^3^
Absorption coefficient	0.809 mm^−1^	0.108 mm^−1^	0.941 mm^−1^	0.119 mm^−1^
F (000)	960	520	696	348
Crystal size	0.20 × 0.20 × 0.10 mm^3^	0.150 × 0.100 × 0.070 mm^3^	0.197 × 0.098 × 0.051 mm^3^	0.150 × 0.110 × 0.050 mm^3^
Theta range for data collection	2.115 to 73.241°	1.581 to 28.938°	4.105 to 73.021°	2.166 to 28.924°
Reflections collected	25595	19572	5533	9647
Independent reflections	4332 [R(int) = 0.0245]	5571 [R(int) = 0.0361]	1604 [R(int) = 0.0198]	3419 [R(int) = 0.0334]
Completeness to theta	67.684° to 98.8%	1.581 to 28.938°	67.684° to 99.8%	2.166 to 28.924°
Refinement method	Full-matrix least-squares on F^2^	Full-matrix least-squares on F^2^	Full-matrix least-squares on F^2^	Full-matrix least-squares on F^2^
Data/restraints/parameters	4332/0/308	5571/0/320	1604/0/145	3419/0/219
Goodness-of-fit on F^2^	0.997	1.073	1.075	1.027
Final R indices [I > 2sigma(I)]	R_1_ = 0.0643, wR_2_ = 0.1510	R_1_ = 0.0457, wR_2_ = 0.1079	R_1_ = 0.0449, wR_2_ = 0.1185	R_1_ = 0.0487, wR_2_ = 0.1038
R indices (all data)	R_1_ = 0.0665, wR_2_ = 0.1518	R_1_ = 0.0695, wR_2_ = 0.1299	R_1_ = 0.0520, wR_2_ = 0.1233	R_1_ = 0.0709, wR_2_ = 0.1135
Extinction coefficient	n/a	n/a	n/a	n/a
Largest diff. peak and hole	0.317 and −0.343 e.Å^−3^	0.310 and −0.289 e.Å^−3^	0.236 and −0.238 e.Å^−3^	0.299 and −0.274 e.Å^−3^
